# CLABSI Reduction Strategies in a Cardiovascular ICU

**DOI:** 10.1097/pq9.0000000000000691

**Published:** 2024-02-21

**Authors:** Heidi Shafland, Lia Johnson, Nicole Johnson, Sarah Murphy

**Affiliations:** From the * Cardiovascular Intensive Care Unit, Children’s MN, Minneapolis, Minn.; † Value and Clinical Excellence, Children’s MN, Minneapolis, Minn.

## INTRODUCTION

Central line blood stream infections (CLABSI) lead to multiple adverse outcomes, including increased morbidity and mortality, procedures, antibiotic use, and cost.^1^ The pediatric cardiac population is uniquely at high risk for CLABSIs due to high acuity and other risk factors, including poor perfusion, increased risk for gut translocation, and recent open-heart surgery. In 2021, the Cardiovascular Intensive Care Unit (CVICU) CLABSI rate was 2.0 (per 1000 central line days). Common themes identified from 2021 to 2022 CLABSI events were the need for vascular access, frequent line access, gut translocation, risk for external contamination from stool or emesis, and patient age of less than 8 months. The CLABSI rate in the CVICU remained the highest in the hospital and showed continued growth over the past 10 years, despite multiple interventions.

## OBJECTIVES

The project’s goal was to reduce the CLABSI rate in the CVICU by standardizing line access procedures and increasing awareness of CLABSI prevention strategies.

## METHODS

A multidisciplinary group of key stakeholders was formed to identify areas of opportunity regarding line maintenance. This group met weekly for 2 months, then monthly thereafter. Strategies were developed to enhance nurse competency, characterize CLABSI risk, reduce unnecessary central lines, and institute new products to reduce infection risk at the insertion site and minimize line access.

Nurses from the CLABSI team were trained to observe line maintenance activities and provide peer-to-peer coaching while promoting psychological safety. The CLABSI team identified variation in staff line maintenance practices and designed education to address practice variations through deliberate practice opportunities, didactic learning, bulletin boards, newsletters, and practice updates.

Multidisciplinary daily rounds were improved to include a focused discussion on the function, utilization and necessity of any intravenous line. Weekly central line rounds were performed on day and night shift, to identify known risk factors for CLABSIs, coach bedside staff to desired standard practices, and facilitate discussion of high-risk patients with the entire medical team.

Two products were introduced to help decrease CLABSI risk. A closed med-line system for intermittent medications was adopted to decrease line access. Additionally, tissue adhesive approved for application at vascular access sites was also adopted to decrease infection risk at the insertion site and maintain dressing integrity.

## RESULTS

**Fig. 1.**
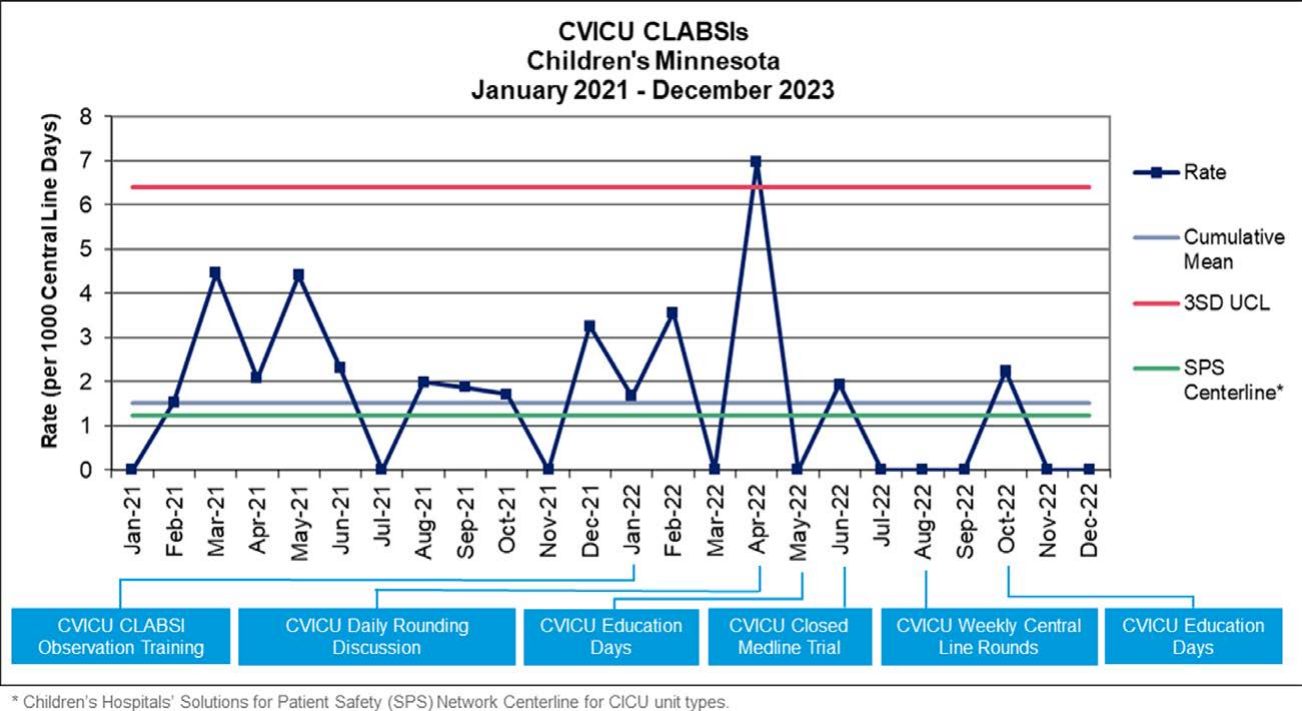
Children's Minnesota CVICU CLABSI rates from 2021 to 2022. Statistical process control chart showing quarterly central line associated bloodstream infection (CLABSI) rates from 2021 through 2022. Includes Children’s Hospitals’ Solutions for Patient Safety (SPS) Network centerline for Cardiovascular Intensive Care Unit (CICU) types as a comparison to like units. #Three standard deviation upper control limit (3 SD UCL). *Children’s Hospitals’ SPS Network Centerline for Cardiovascular Intensive Care Unit (CICU) types.

Since implementing the above measures, the CLABSI rate decreased 24% from 2021 to 2022 (Fig. 1). In the first 6 months of 2022, eight CLABSIs were identified with a rate of 2.42, and in the last six months of 2022, one CLABSI was identified with a rate of 0.39 (Table 1). The closed med-line system decreased line access by 50%.

**Table 1.** Monthly CLABSI numbers and rates for CVICU at Children's Minnesota during 2022

Month	CVICU CLABSI Total	Rate(Per 1000 Central Line Days)	
Jan 2022	1	1.68	2.42
Feb 2022	2	3.57	
Mar 2022	0	0.00	
Apr 2022	4	6.98	
May 2022	0	0.00	
Jun 2022	1	1.94	
Jul 2022	0	0.00	0.39
Aug 2022	0	0.00	
Sep 2022	0	0.00	
Oct 2022	1	2.25	
Nov 2022	0	0.00	
Dec 2022	0	0.00	

Rates are shown per 1,000 central line days and are broken down by month and 6-month periods.

## CONCLUSIONS

Our results show multidisciplinary engagement in unit-wide initiatives resulted in better compliance with CLABSI reduction interventions. Regular and consistent CLABSI prevention rounding practices, ongoing education, and identification of patients with CLABSI risk factors were key to increased awareness, improved standard practice, and overall decreased CLABSI rate. Successfully implementing high-impact sustainable interventions have motivated improvements in other units throughout the organization.
